# Motor performance and 3D gait pattern characteristics in older adult women with early mild cognitive impairment: a pilot study

**DOI:** 10.3389/fnagi.2026.1815595

**Published:** 2026-06-22

**Authors:** Ho-Jong Gil, Jongbin Kim

**Affiliations:** 1 Institute of Sport Science, Korea National Sport University, Seoul, Republic of Korea; 2 Graduate School of Education, Silla University, Busan, Republic of Korea

**Keywords:** early mild cognitive impairment, gait analysis, lower-limb kinematics, older women, postural balance

## Abstract

**Introduction:**

Early mild cognitive impairment (eMCI) is often accompanied by subtle alterations in motor function and gait control before the onset of overt dementia. Identifying these early functional changes may provide useful information for early detection and intervention. This study compared motor performance, balance, gait characteristics, and lower-limb kinematics between older women with eMCI and cognitively healthy controls.

**Methods:**

Thirty-four community-dwelling older women participated in this exploratory pilot study, including 17 women with eMCI and 17 age-matched healthy controls. Body composition was assessed using a bioelectrical impedance analyzer. Physical fitness was evaluated using the Senior Fitness Test, including grip strength, the 30-second Chair Stand Test, and the Figure-of-Eight Walk Test. Static balance was assessed using center-of-pressure variables, and gait characteristics and lower-limb kinematics were measured using a three-dimensional gait analysis system at a standardized walking speed of 1.0 m/s. Group differences were analyzed using the Mann-Whitney *U*-test, and effect sizes were calculated.

**Results:**

No significant between-group differences were observed in body composition or grip strength (*p* > 0.05). However, the eMCI group demonstrated significantly lower performance in the 30-second Chair Stand Test (*p* < 0.001). In balance assessment, total center-of-pressure path length was significantly greater, whereas sway velocity was significantly lower in the eMCI group (*p* < 0.05). In gait analysis, stride length, gait cycle time, and double-limb support time were significantly greater in the eMCI group (*p* < 0.001). Furthermore, women with eMCI exhibited reduced right knee joint range of motion in the frontal and transverse planes and lower right hip angular velocity in the frontal plane (*p* < 0.05).

**Discussion:**

Older women with early mild cognitive impairment exhibited subtle but meaningful impairments in lower-limb strength, postural control, gait performance, and joint-level motor control despite relatively preserved body composition and basic gait ability. These findings suggest that detailed balance and gait-related kinematic variables may serve as sensitive indicators of early motor-control alterations associated with cognitive decline and may contribute to the development of early screening and intervention strategies.

## 1 Introduction

Due to population aging, the prevalence of dementia continues to rise. Despite extensive research, the causes of dementia have still not been clearly elucidated, thereby limiting the development of root-cause treatments (Kim et al., 2014). As a result, the economic burden associated with dementia is steadily increasing, alongside growing personal and societal costs (Kim and Lee, 2014). Because dementia is difficult to treat once it progresses to a severe stage, early detection and intervention during early mind cognitive impairment (MCI) or early-stage dementia are critically important (Kim et al., 2017).

Early diagnosis is particularly challenging because the initial symptoms of dementia are similar to those of MCI and age-related forgetfulness. Therefore, early detection and intervention at the MCI stage have emerged as key strategies for dementia prevention (Oh and Lee, 2016; Lin et al., 2012). Individuals with MCI present with some cognitive dysfunction without major restrictions in activities of daily living, and timely intervention at this stage may delay or mitigate progression to dementia (Weissberger et al., 2018). Although objective brain imaging examinations—such as computed tomography (CT), magnetic resonance imaging (MRI), and positron emission tomography (PET)—are used for early diagnosis of MCI and dementia, their application is difficult to perform regularly due to several factors, such as the need for specialized personnel, the time required, and the cost (Graff-Radford et al., 2021).

Cognitive dysfunction has also been closely associated with physical fitness factors. In particular, reduced upper and lower limb muscle strength has been linked to decreased cognitive function (Kim et al., 2013). Cardiovascular endurance and flexibility have also shown significant correlations with cognitive function (In-Hwan and Hyun-Sik, 2016). Previous research indicates that regular physical activity and structured exercise programs can improve cognitive function, reduce dementia risk factors in older adults (Northey et al., 2018; Erickson et al., 2019; Stillman et al., 2020), and support independent.

Taken together, exercise participation among older adult women with MCI may play a crucial role in promoting healthy aging by improving cognitive function, enhancing physical fitness, fostering positive psychological states, and preventing chronic disease. In the present study, motor performance was evaluated with a focus on functional fitness elements directly related to gait, including lower limb muscle strength, balance, and transfer ability. These components are closely associated with gait performance; therefore, changes in these indicators may reflect broader alterations in overall motor function. Accordingly, gait assessment may provide a useful behavioral measure of motor performance.

Recently, gait assessment has gained interest as a simple and objective measure of cognitive function (Yang et al., 2020). Compared with neuroimaging, gait assessment is more cost-effective and accessible and can be naturally measured during daily activities, making it a potentially useful approach for identifying mobility-related changes associated with MCI. Cognitive dysfunction, particularly involving the frontal lobe and hippocampus, may impair information processing, memory storage and recall, and nerve conduction. These deficits can subsequently affect motor control, resulting in reduced gait stability and slower walking speed (Beauchet et al., 2019; Montero-Odasso et al., 2020; Mirelman et al., 2022). Indeed, improvements in gait speed have been observed after the use of dementia treatments such as donepezil and tetrahydrocannabinol (THC; Montero-Odasso et al., 2015; Van Den Elsen et al., 2017).

However, previous studies investigating the relationship between gait and cognitive function have primarily focused on intervention outcomes, with relatively few studies systematically examining differences in gait patterns (Mc Ardle et al., 2019; Del Din et al., 2021; Satt et al., 2020). In addition, most existing studies have concentrated on general spatiotemporal gait parameters, such as walking speed and stride length. In contrast, a more detailed examination of three-dimensional kinematic variables, including joint range of motion and angular velocity, may provide deeper insights into gait control, particularly in older women at the early stage of MCI-W. Recent studies have suggested that gait characteristics, postural control, and digital biomarkers may serve as sensitive indicators of early cognitive decline and age-related functional deterioration in older adults (Bayot et al., 2020; Kwok et al., 2024; Wang et al., 2025). Such an approach enables the detection of subtle alterations in motor control that are not easily captured by conventional gait indicators.

Moreover, early stages of MCI, individual variability in neurological impairment and motor control strategies may be present, making it difficult for large-scale studies alone to fully capture these subtle functional characteristics. Unlike previous studies that have primarily focused on general spatiotemporal gait parameters, this study specifically examines joint-level kinematic variables (e.g., ROM and angular velocity) in older women with early-stage cognitive impairment. By capturing subtle alterations in motor control that are not detectable through conventional gait measures, may provide a complementary and potentially sensitive perspective for identifying subtle motor-control alterations associated with early cognitive impairment. Therefore, the present study aims to comprehensively analyze motor performance and gait pattern characteristics in older adult women in the early stages of MCI and to identify patterns of early functional change. The findings are expected to provide foundational evidence for the development of targeted exercise intervention strategies for this population.

## 2 Methods

### 2.1 Participants

The study included 34 community-dwelling older adult women residing in B metropolitan city, comprising 17 participants with early mild cognitive impairment (eMCI-W) and 17 cognitively healthy controls (HC-W). Participants were recruited from community welfare centers and senior activity centers using a convenience sampling approach. Recruitment flyers describing the study purpose, procedures, and eligibility criteria were distributed and posted at the facilities, and on-site staff assisted in informing potential participants. Interested individuals voluntarily contacted the research team and underwent an initial screening process conducted by trained researchers. Eligibility was assessed through structured interviews, which included verification of age, health status, and mobility based on predefined inclusion and exclusion criteria. All participants had no history of visual, auditory, or cardiovascular disease, had not undergone treatment or surgery for musculoskeletal disorders within the previous 6 months, and were capable of independent ambulation without any assistive devices (e.g., cane, ankle-foot orthosis).

Following recruitment, participants were classified into cognitive groups using a standardized cognitive assessment. Cognitive function was evaluated using the Montreal Cognitive Assessment (MoCA), administered through structured face-to-face interviews by trained evaluators (Malek-Ahmadi et al., 2024; Parra-Rodríguez et al., 2025). The MoCA is a widely used screening tool for detecting mild cognitive impairment, with a maximum score of 30 points. A cutoff score of < 26 was used to identify individuals with cognitive impairment, in accordance with established guidelines. Participants who demonstrated cognitive impairment based on the MoCA while maintaining independence in activities of daily living were classified as having MCI, consistent with Petersen's criteria. In contrast, individuals with MoCA scores within the normal range (≥26) and without cognitive complaints were classified as cognitively healthy controls. This classification process was conducted to ensure consistent and systematic differentiation between groups prior to physical and gait assessments. This classification was based on a screening approach using MoCA scores and did not constitute a formal clinical diagnosis. Therefore, the possibility of misclassification and heterogeneity within the cognitive impairment group cannot be excluded.

Prior to participation, all participants received a comprehensive explanation of the study procedures, including measurement protocols, potential risks, and expected benefits. Written informed consent was obtained from all participants. The study was approved by the Institutional Review Board (IRB No. IRB1041449-202302-HR-001). The participants' physical characteristics are summarized in Table 1. Because this study was designed as an exploratory pilot investigation, a priori power calculation was not performed. Instead, the primary purpose was to estimate preliminary effect sizes and variability for future adequately powered longitudinal studies.

**Table 1:** Participant characteristics.

Group	eMCI-W (*n* = 17)	HC-W (*n* = 17)	*p*-value
Age (yrs)	69.47 (66.90–72.04)	69.17 (67.41–70.93)	0.759
Height (m)	165.00 (158.50–173.75)	165.00 (162.00–173.00)	0.754
Weight (kg)	61.50 (54.42–68.28)	63.90 (56.00–75.20)	0.509

Values are presented as median (interquartile range). Group differences were analyzed using the Mann–Whitney *U* test.

### 2.2 Study procedures

This pilot study aimed to obtain preliminary data to inform the design of a future large-scale investigation. The sample size was considered appropriate for this exploratory pilot study, as its primary aim was to estimate preliminary effect sizes and variability to inform future large-scale studies, rather than to achieve definitive statistical power. It was designed as an exploratory analysis of physical characteristics, cognitive function, fitness elements, gait patterns, and balance in older women with and without early mild cognitive impairment (eMCI-W). Before measurements, participants were asked to remove any metal accessories that could interfere with data collection. All study procedures were conducted after receiving informed consent.

#### 2.2.1 Physical measurements and cognitive ability

Physical characteristics were assessed using a body composition analyzer (InBody 270, Co., Ltd., Seoul, Korea). As this device does not directly measure height, height was measured separately using a standard stadiometer (Seca 213, Seca GmbH & Co. KG, Hamburg, Germany), measuring height (cm), weight (kg), body fat percentage (%Fat), body mass index (BMI, kg/m^2^), total muscle mass (kg), and skeletal muscle mass (kg). Initial cognitive function was evaluated using the Montreal Cognitive Assessment (MoCA), originally developed by (Nasreddine et al. 2005). The MoCA is a brief cognitive screening tool designed to detect mild cognitive impairment and consists of multiple domains, including attention, executive function, memory, language, visuospatial ability, abstraction, calculation, and orientation. The total score ranges from 0 to 30 points, with higher scores indicating better cognitive function. A cutoff score of < 26 was used to identify individuals with cognitive impairment, in accordance with established guidelines. The MoCA has been shown to have high sensitivity and reliability for detecting MCI in both clinical and research settings (Nasreddine et al., 2005; Malek-Ahmadi et al., 2024; Parra-Rodríguez et al., 2025). In the present study, internal consistency reliability was acceptable, with a Cronbach's α of 0.81. Participants were classified as having eMCI-W based on MoCA scores (< 26) and the absence of functional impairment in activities of daily living, consistent with Petersen's criteria. Participants with suspected dementia or diagnosed neurological disorders were excluded.

#### 2.2.2 Senior fitness test analysis

To ensure the reliability and validity of the Senior Fitness Test (SFT), the second edition (2014) of the Korean translation of the Senior Fitness Test Manual of Rikli (2013) was followed. Standardized testing instruments were used for all assessment items, and examiners completed 10 training sessions over 4 weeks. Examiners were additionally trained in safety and prevention measures applicable throughout the testing process. The SFT was divided into upper and lower limb strength.

**Figure 1:**
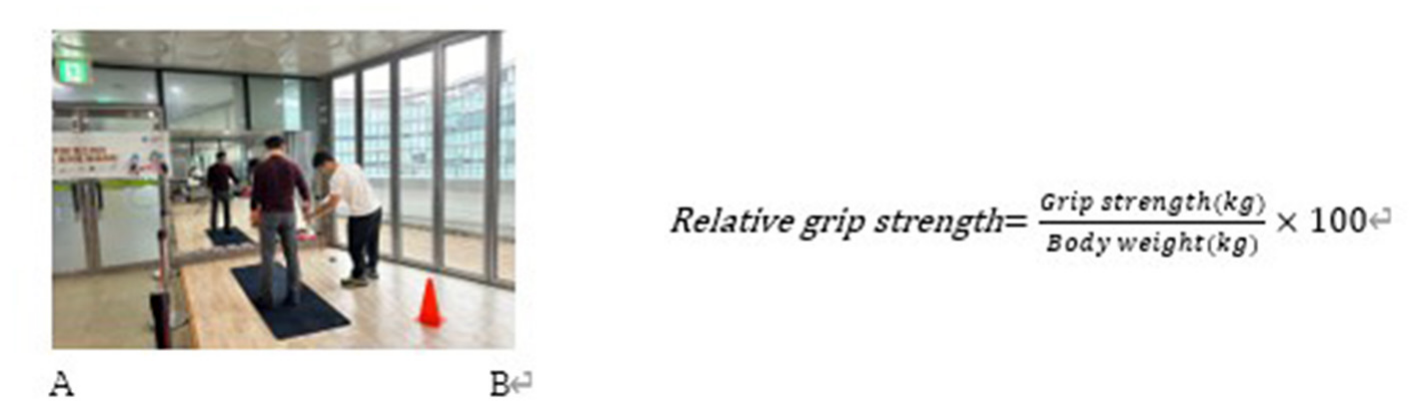
**(A)** Posture for relative grip strength measurement and **(B)** formula.

**Figure 2:**
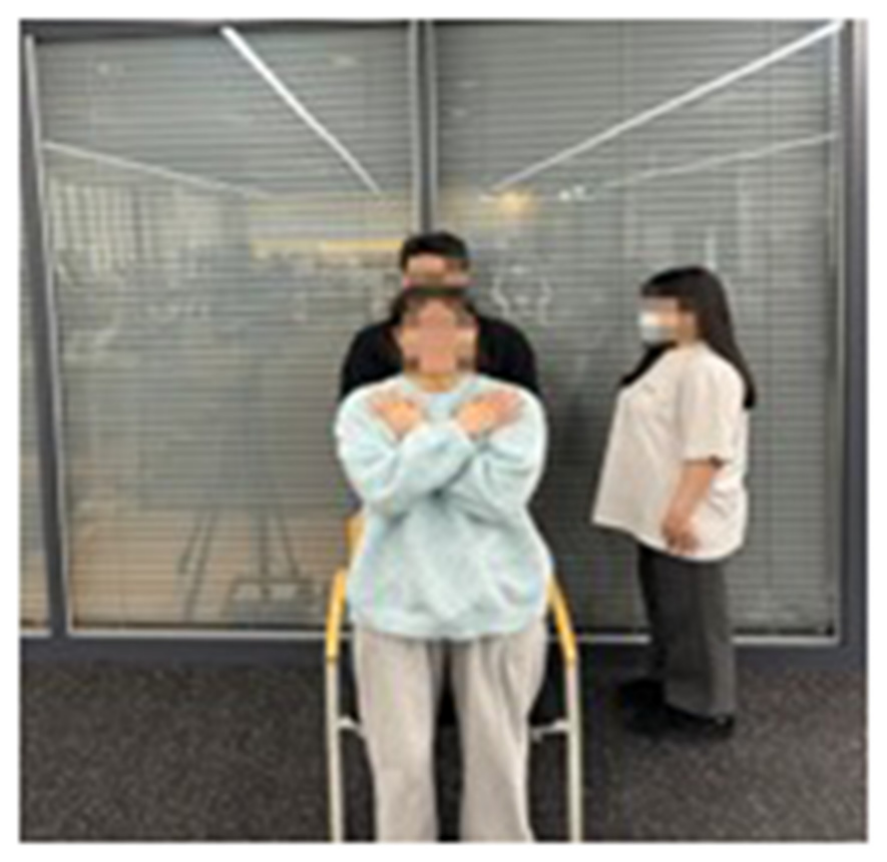
30-second Chair Stand Test posture for assessing lower limb muscle strength.

**Figure 3:**
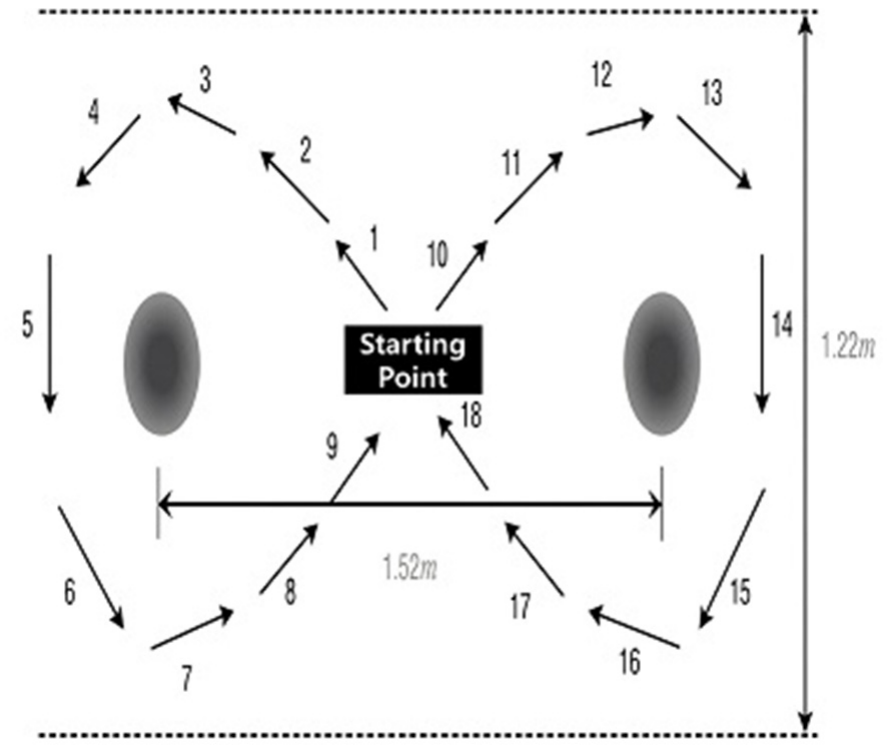
Figure-of-Eight Walk test.

Upper limb strength was measured using a hand dynamometer (T.K.K.5401, Takei, Japan). Grip strength was measured twice for each hand, and the average measurement was recorded and used to calculate the relative grip strength (%; Figure 1). Lower limb strength was assessed using the 30-second Chair Stand Test (CS-30), which records the number of full sit-to-stand repetitions completed within 30 s (Figure 2). The 30-second Chair Stand Test was performed to assess lower limb muscle strength following the standardized protocol of the Senior Fitness Test. The chair used in this test had a seat height of 43 cm, with a firm, flat surface and no armrests. Participants were seated in the middle of the chair with their back straight, feet flat on the floor at shoulder-width apart, and arms crossed over the chest. Upon the “go” signal, participants were instructed to stand up to a full upright position and then return to a seated position as many times as possible within 30 s. A full standing position was defined as complete hip and knee extension, and a valid repetition was counted only when the participant returned to a fully seated position with the hips in contact with the chair. All repetitions were counted by a trained evaluator using a standardized counting procedure, and verbal encouragement was provided to maintain maximal effort. One familiarization trial was allowed prior to the test to ensure understanding of the procedure. Finally, the Figure-of-Eight Walk Test was performed to measure agility and dynamic balance. After thoroughly explaining the procedure, participants completed two trials, and the shortest time was used as the representative value. The order of the SFT components was randomized for each participant (Figure 3).

#### 2.2.3 Balance assessment

Static balance was measured using the Gaitview AFA-50 system (AlFOOT, Korea). Participants stood barefoot on the measurement device with their feet positioned a shoulder-width apart. While focusing on a fixed point located 2 m ahead, participants maintained a static posture for 30 s. During this period, the center of pressure (COP) displacement data were recorded.

**Figure 4:**
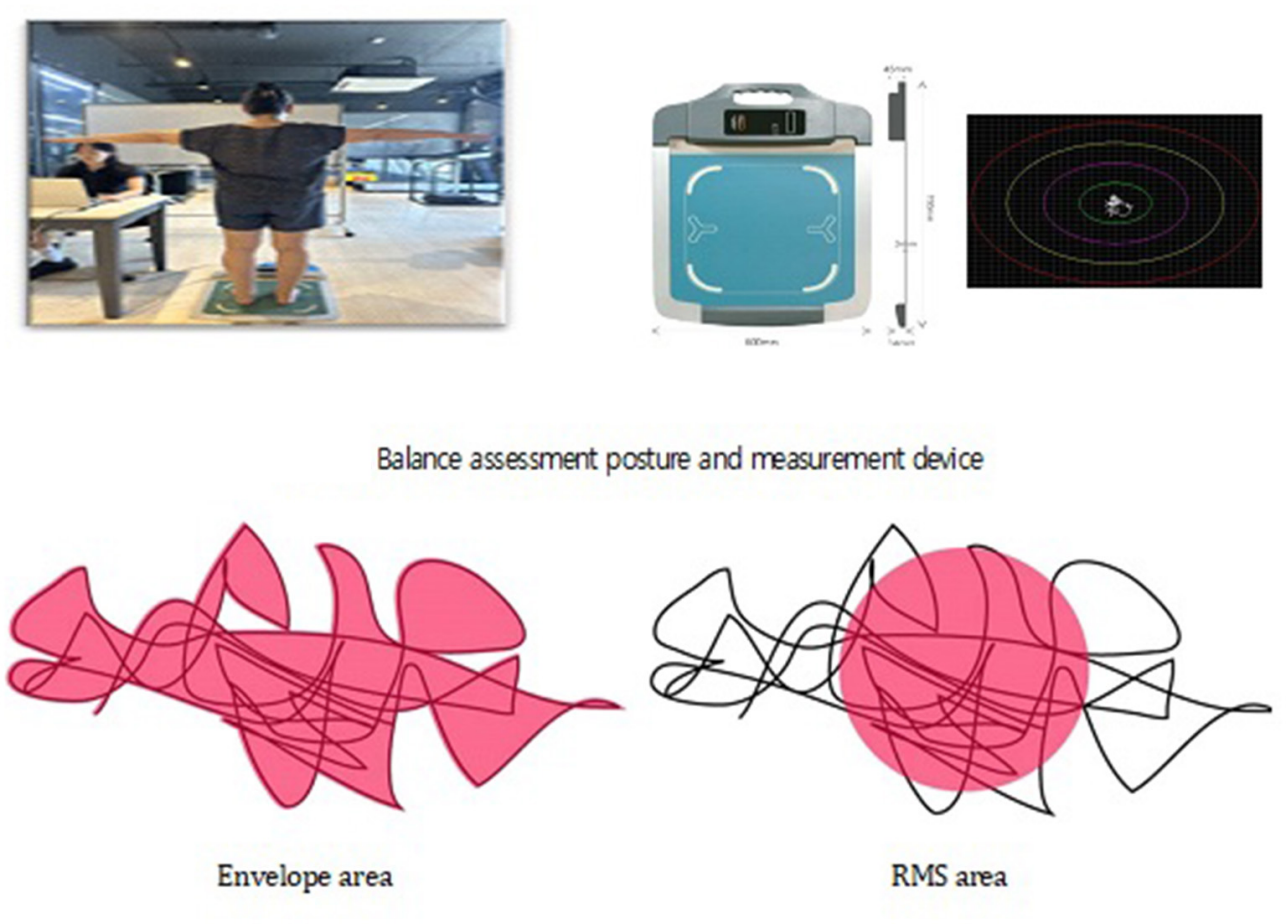
Balance assessment posture and measurement device.

The following parameters were analyzed (Figure 4):

- Envelope area (ENV): The area of the outermost trajectory of the COP path.
- Root mean square (RMS): The dispersion of COP displacement relevant to the mean COP position, represented as a circular area calculated via algorithmic modeling.
- Total path length: The total distance traveled by the COP during the measurement period.
- COP sway velocity: The total path length divided by the test duration.

Lower values for these parameters indicate better balance. In this study, the ENV, RMS, total path length, and sway velocity were analyzed.

#### 2.2.4 Gait pattern analysis

**Figure 5:**
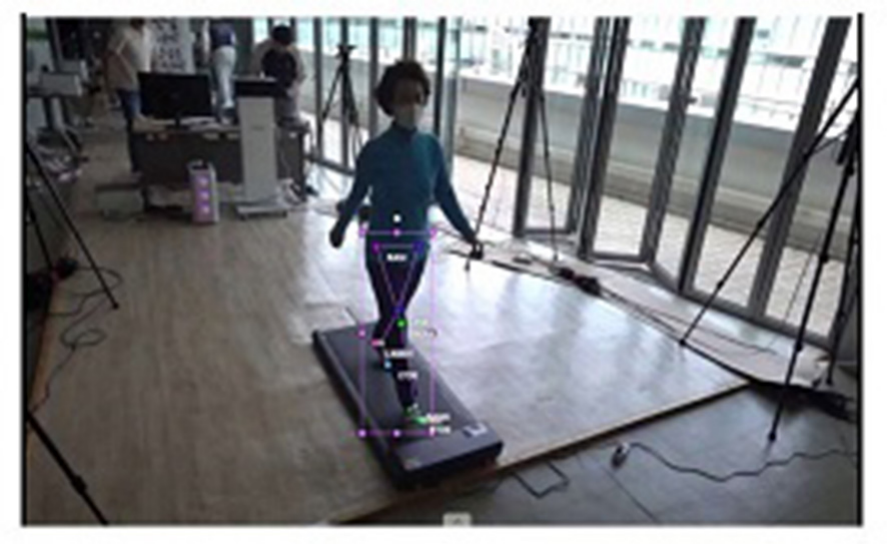
Gait pattern measurement.

Quantitative gait analysis was performed using a musculoskeletal gait analysis device (Clinical Gait Analysis, 6100 RMT, Exbody, Seoul, Korea) to measure spatiotemporal gait parameters (Figure 5; Beauchet et al., 2019). The analyzed spatiotemporal variables included stride length (m), stride width (m), gait cycle time (s), and double-limb support time (s). Walking speed was standardized at 1.0 m/s, as predetermined by the researchers (He et al., 2025). A gait cycle was defined, using the right foot as reference, as the interval between initial heel contact (HC) and the subsequent HC of the same foot. Within this cycle, the stance phase was defined as the period from HC to toe-off (TO), and the swing phase was defined as the time from TO to HC.

#### 2.2.5 Joint range of motion and angular velocity analysis during gait

Kinematic variables included flexion-extension and rotational joint angles (°) of the neck, torso, hip, and knee. Postural and pelvic stability variables included lateral tilt of the torso (°), pelvic lateral displacement (mm), pelvic vertical displacement (mm), and pelvic rotation (°). For gait ability testing, participants stood on the gait analysis device and began walking at a speed of 1.0 m/s upon verbal instruction from the examiner. Participants wore comfortable shoes during testing. Each trial was repeated three times under identical conditions, and the mean measurements at rest were used for analysis. The definitions used for the angles of the lower limb joints were: *X*, flexion–extension (sagittal plane); *Y*, ab/adduction (frontal plane); and *Z*, internal–external rotation (transverse plane).

### 2.3 Statistical processing

Statistical analyses were conducted using IBM SPSS Statistics version 26.0 (IBM Corp., Armonk, NY, USA). Descriptive statistics are presented as median and interquartile range (IQR) for all variables. Data normality was assessed using the Shapiro–Wilk test. As several variables did not satisfy the assumption of normality, between-group differences were analyzed using the non-parametric Mann–Whitney *U* test. Statistical significance was set at *p* < 0.05. Effect size (*r*) was calculated by dividing the standardized test statistic (*Z*) by the square root of the total sample size (*N*). Effect sizes were interpreted as small (*r* ≥ 0.10), medium (*r* ≥ 0.30), and large (*r* ≥ 0.50).

## 3 Results

This was an exploratory pilot study to compare body composition, physical fitness (SFT), static balance, gait patterns, and lower limb range of motion (ROM) and angular velocity between the eMCI-W and HC-W groups.

### 3.1 Body composition and SFT comparison

In the comparison of body composition variables, BMI did not differ significantly between the eMCI-W group (22.60 [18.00–27.20] kg/m^2^) and the HC-W group (23.47 [18.70–28.70] kg/m^2^; *U* = 150.5, *p* = 0.947, and *r* = 0.06) (Table 2). Similarly, no significant differences were found in body fat percentage (*U* = 142.5, *p* = 0.741, and *r* = 0.06) or muscle mass (*U* = 151.5, *p* = 0.970, and *r* = 0.01). In the SFT variables, grip strength showed no significant differences between groups for both the left (*U* = 159.5, *p* = 0.843, and *r* = 0.04) and right hands (*U* = 170.5, *p* = 0.575, and *r* = 0.10). However, a significant difference was observed in the 30-second Chair Stand Test, with the eMCI-W group (22.50 [18.75–28.25] repetitions) showing lower performance compared to the HC-W group (28.00 [20.00–40.00] repetitions; *U* = 43.5, *p* < 0.001, and *r* = 0.61). Similarly, the Figure-of-Eight Walk Test did not show a significant difference between groups (*U* = 120.0, *p* = 0.403, and *r* = 0.15).

**Table 2:** Comparison of body composition and fitness (SFT) between the eMCI-W and HC-W groups.

Variables	eMCI-W	HC-W	*U*	*p*-value	Effect
	median (IQR)	median (IQR)			size (*r*)
BMI (kg/m^2^)	22.60 (18.00–27.20)	23.47 (18.70–28.70)	150.5	0.947	0.06
Body fat percentage (%)	25.95 (17.40–31.35)	26.50 (22.70–30.80)	142.5	0.741	0.06
Muscle mass (kg)	43.80 (36.98–51.27)	42.50 (38.60–51.70)	151.5	0.970	0.01
Grip strength – *L* (%)	29.85 (22.48–42.88)	29.60 (23.00–37.60)	159.5	0.843	0.04
Grip strength – *R* (%)	30.70 (23.00–42.50)	30.10 (23.60–37.50)	170.5	0.575	0.10
30-second Chair Stand Test (reps)	22.50 (18.75–28.25)	28.00 (20.00–40.00)	43.5	< 0.001	0.61
Figure-of-Eight Walk test (s)	32.80 (28.70–34.23)	30.42 (27.38–34.52)	120.0	0.403	0.15

eMCI-W, women with early cognitive impairment; HC-W, healthy control women.

Values are presented as median (interquartile range). Group differences were analyzed using the Mann–Whitney *U* test.

### 3.2 Balance analysis

In the static balance analysis, no significant differences were observed between the eMCI-W and HC-W groups in ENV (*U* = 169.0, *p* = 0.609, and *r* = 0.09) or RMS (*U* = 138.0, *p* = 0.632, and *r* = 0.08) (Table 3). However, total path length was significantly greater in the eMCI-W group (209.95 [87.90–337.20] mm) compared to the HC-W group (108.40 [68.90–135.40] mm; *U* = 228.0, *p* = 0.014, and *r* = 0.42). In contrast, COP sway velocity was significantly lower in the eMCI-W group (5.25 [3.80–5.78] mm/s) than in the HC-W group (8.00 [6.90–9.20] mm/s; *U* = 40.5, *p* < 0.001, and *r* = 0.63).

**Table 3:** Comparison of balance between the eMCI-W and HC-W groups.

Variables	eMCI-W	HC-W	*U*	*p*-value	Effect
	median (IQR)	median (IQR)			size (*r*)
ENV (mm^2^)	56.00 (34.78–103.72)	53.80 (39.50–63.20)	169.0	0.609	0.09
RMS (mm^2^)	34.40 (18.90–47.05)	35.60 (19.30–72.90)	138.0	0.632	0.08
Total Length (mm)	209.95 (87.90–337.20)	108.40 (68.90–135.40)	228.0	**0.014**	0.42
COP sway velocity (mm/s)	5.25 (3.80–5.78)	8.00 (6.90–9.20)	40.5	**0.001**	0.63

eMCI-W, women with early cognitive impairment; HC-W, healthy control women; ENV, Envelope area; RMS, Root mean square; COP, center of pressure.

Values are presented as median (interquartile range). Group differences were analyzed using the Mann–Whitney *U* test. Bold values indicate statistically significant differences between groups (*p* < 0.05).

### 3.3 Gait pattern analysis

In the analysis of gait patterns, several spatiotemporal parameters showed significant differences between the eMCI-W and HC-W groups (Table 4). Stride length was significantly greater in the eMCI-W group (1.05 [1.05–1.07] m) compared to the HC-W group (1.03 [1.02–1.03] m; *U* = 242, *p* < 0.001, and *r* = 0.58). Similarly, cycle time was significantly longer in the eMCI-W group (0.96 [0.95–0.98] s) than in the HC-W group (0.94 [0.93–0.94] s; *U* = 242, *p* < 0.001, and *r* = 0.58). Double limb support time was also significantly greater in the eMCI-W group (0.29 [0.29–0.29] s) compared to the HC-W group (0.27 [0.27–0.27] s; *U* = 240, *p* < 0.001, and *r* = 0.60). In contrast, stride width did not show a statistically significant difference between the groups (*U* = 191, *p* = 0.100, and *r* = 0.28).

**Table 4:** Comparison of gait patterns between the eMCI-W and HC-W groups.

Variables	eMCI-W	HC-W	*U*	*p*-value	Effect
	median (IQR)	median (IQR)			size (*r*)
Stride length (m)	1.05 (1.05–1.07)	1.03 (1.02–1.03)	242	0.0**01**	0.58
Stride width (m)	0.12 (0.11–0.12)	0.10 (0.10–0.11)	191	0.100	0.28
Cycle time (s)	0.96 (0.95–0.98)	0.94 (0.93–0.94)	242	**0.001**	0.58
Double limb support (s)	0.29 (0.29–0.29)	0.27 (0.27–0.27)	240	**0.001**	0.60

eMCI-W, women with early cognitive impairment; HC-W, healthy control women.

Values are presented as median (interquartile range). Group differences were analyzed using the Mann–Whitney *U* test. Bold values indicate statistically significant differences between groups (*p* < 0.05).

### 3.4 Analysis of lower limb joint angles

In the analysis of lower limb joint ROM, most variables showed no statistically significant differences between the eMCI-W and HC-W groups (Table 5). For the right lower limb, no significant differences were observed in ankle joint ROM across all axes (*X, Y*, and *Z*; *p* > 0.05), and knee joint ROM in the sagittal plane (*X* axis) also showed no significant difference (*p* = 0.92). However, significant differences were found in the knee joint ROM in the frontal (*Y* axis; *U* = 106.0, *p* = 0.02, and *r* = 0.59) and transverse planes (*Z* axis; *U* = 119.5, *p* = 0.04, and *r* = 0.54), with the eMCI-W group showing lower values compared to the HC-W group. For the hip joint, no significant differences were found in any axis for the right side (*p* > 0.05). For the left lower limb, no statistically significant differences were observed in ankle or knee joint ROM across all axes (*p* > 0.05). In the hip joint, there was no significant difference in the sagittal or transverse planes (*p* > 0.05), although a non-significant trend was observed in the frontal plane (*Y* axis; *U* = 131.0, *p* = 0.07, and *r* = 0.38).

**Table 5:** Comparison of lower limb ROM between the eMCI-W and HC-W groups.

Variables			eMCI-W	HC-W	*U*	*p*-value	Effect size (*r*)
RoM			median (IQR)	median (IQR)			
Right	ANKLE	*X*	27.02 (23.22–30.82)	25.83 (19.04–32.62)	242.0	0.747	0.08
		*Y*	9.39 (7.17–11.61)	9.47 (6.82–12.12)	213.0	0.843	0.05
		*Z*	12.97 (11.27–14.67)	12.97 (9.34–16.60)	238.0	0.801	0.05
	KNEE	*X*	42.41 (39.25–45.57)	42.41 (35.74–49.08)	230.0	0.926	0.01
		*Y*	2.92 (1.81–4.03)	4.55 (2.67–6.43)	106.0	**0.022**	0.59
		*Z*	0.04 (0.02–0.06)	0.05 (0.02–0.08)	119.5	**0.043**	0.54
	HIP	*X*	38.48 (34.28–42.68)	39.28 (33.03–45.53)	197.0	0.622	0.10
		*Y*	5.39 (3.55–7.23)	6.64 (4.58–8.70)	167.0	0.285	0.37
		*Z*	7.88 (6.49–9.27)	7.34 (5.28–9.40)	255.0	0.567	0.13
Left	ANKLE	*X*	26.25 (21.45–31.05)	26.24 (20.34–32.14)	227.0	0.968	0.01
		*Y*	10.20 (7.49–12.91)	10.76 (7.85–13.67)	201.0	0.671	0.10
		*Z*	14.53 (10.09–18.97)	13.04 (10.16–15.92)	286.0	0.241	0.14
	KNEE	*X*	40.85 (38.27–43.43)	40.45 (35.11–45.79)	229.0	0.936	0.01
		*Y*	7.05 (4.63–9.47)	6.63 (3.78–9.48)	264.0	0.455	0.29
		*Z*	7.52 (6.15–8.89)	6.82 (4.84–8.80)	275.0	0.346	0.30
	HIP	*X*	41.76 (37.51–46.01)	42.29 (36.66–47.92)	213.0	0.847	0.05
		*Y*	3.07 (2.30–3.84)	4.09 (2.50–5.68)	131.0	0.077	0.38
		*Z*	0.06 (0.03–0.09)	0.06 (0.02–0.10)	267.5	0.418	0.19

eMCI-W, women with early cognitive impairment; HC-W, healthy control women.

X, flexion–extension (sagittal plane); Y, ab/adduction (frontal plane); Z, internal–external rotation (transverse plane).

Values are presented as median (interquartile range). Group differences were analyzed using the Mann–Whitney *U* test. Bold values indicate statistically significant differences between groups (*p* < 0.05).

### 3.5 Lower limb angular velocity analysis

In the analysis of lower limb joint angular velocity, most variables showed no statistically significant differences between the eMCI-W and HC-W groups (Table 6). For the lower right limb, no significant differences were observed in ankle and knee joint angular velocity across all axes (*p* > 0.05). However, a significant difference was found in the hip joint angular velocity in the frontal plane (*Y* axis), with the eMCI-W group (30.41 [21.12–39.70] °/s) showing significantly lower values compared to the HC-W group (52.30 [35.05–69.55] °/s; *U* = 56.0, *p* < 0.001, and *r* = 0.61). For the left lower limb, no statistically significant differences were observed in ankle or knee joint angular velocity across all axes (*p* > 0.05). Similarly, hip joint angular velocity did not differ significantly between groups in any plane (*p* > 0.05).

**Table 6:** Comparison of lower limb joint angular velocity between the eMCI-W and HC-W groups.

Variables			eMCI-W	HC-W	*U*	*p*-value	Effect size (*r*)
Angular velocity (°/s)			median (IQR)	median (IQR)			
Right	ANKLE	*X*	286.63 (254.76–318.50)	292.50 (234.17–350.83)	209.0	0.780	0.05
		*Y*	74.93 (59.27–90.59)	90.38 (58.06–122.70)	139.0	0.103	0.23
		*Z*	185.66 (142.29–229.03)	182.70 (122.33–243.07)	228.0	0.955	0.03
	KNEE	*X*	284.69 (237.00–332.38)	307.18 (253.27–361.09)	167.0	0.283	0.20
		*Y*	88.44 (59.84–117.04)	81.59 (47.38–115.80)	264.0	0.456	0.16
		*Z*	93.38 (77.96–108.80)	89.52 (52.15–126.89)	252.0	0.604	0.13
	HIP	*X*	370.95 (321.06–420.84)	402.79 (305.82–499.76)	162.0	0.247	0.24
		*Y*	30.41 (21.12–39.70)	52.30 (35.05–69.55)	56.0	**0.001**	0.61
		*Z*	13.48 (10.26–16.70)	18.84 (13.34–24.34)	170.0	0.316	0.20
Left	ANKLE	*X*	290.72 (264.06–317.38)	295.43 (243.82–347.04)	201.0	0.674	0.07
		*Y*	88.20 (63.78–112.62)	93.04 (70.23–115.85)	208.0	0.774	0.05
		*Z*	182.10 (121.15–243.05)	188.37 (135.46–241.28)	228.0	0.958	0.02
	KNEE	*X*	305.38 (273.72–337.04)	314.32 (279.68–348.96)	200.0	0.667	0.10
		*Y*	94.25 (69.68–118.82)	80.07 (47.39–112.75)	297.0	0.172	0.28
		*Z*	90.92 (72.78–109.06)	87.07 (66.00–108.14)	258.0	0.536	0.15
	HIP	*X*	378.92 (323.17–434.67)	418.18 (322.95–513.41)	150.0	0.164	0.29
		*Y*	43.74 (30.35–57.13)	48.54 (34.68–62.40)	170.0	0.315	0.20
		*Z*	12.70 (1.60–23.80)	18.36 (6.89–29.83)	142.0	0.121	0.30

eMCI-W, women with early cognitive impairment; HC-W, healthy control women.

X, flexion–extension (sagittal plane); Y, ab/adduction (frontal plane); Z, internal–external rotation (transverse plane). Values are presented as median (interquartile range). Group differences were analyzed using the Mann–Whitney *U* test. Bold values indicate statistically significant differences between groups (*p* < 0.05).

## 4 Discussion

In this study, physical characteristics, functional fitness, gait patterns, balance, ROM, and angular velocity were measured in older women with eMCI-W to examine their associations with cognitive function.

First, no statistically significant differences were observed in BMI, body fat percentage, or muscle mass between the eMCI-W and HC-W groups. These findings suggest that changes in body composition may not be evident during the early stages of cognitive impairment. (Petersen et al. 2014) have reported that BMI and body fat percentage in individuals with MCI are generally comparable to those of healthy older adults, whereas gradual weight loss and decreased muscle mass tend to emerge as dementia advances. Previous studies suggest that body composition changes may be delayed in the early stages of neurodegenerative disease due to relatively preserved metabolic function and physical activity levels. Nevertheless, long-term reductions in muscle mass have been consistently associated with dementia progression. (Delmonico et al. 2009) reported an increased risk of dementia among older adults with sarcopenia, and decreased muscle mass and sarcopenia have been increasingly recognized as risk factors for cognitive decline and Alzheimer's disease (Cruz-Jentoft et al., 2019). Although there were no differences between the two groups in the present study, this is thought to be because the participants were exclusively in the early stage of cognitive impairment. It is plausible that body composition changes become increasingly apparent as cognitive decline progresses. Taken together, these findings indicate that body composition measures may be more appropriately considered as longitudinal or complementary indicators of dementia risk rather than as sensitive markers of early-stage mild cognitive impairment.

Second, in the SFT, although there were no between-group differences in grip strength, the eMCI-W group showed far poorer performance in the 30-second Chair Stand Test (*p* < 0.001). Lower limb muscle strength and endurance are directly related to decreased gait and balance ability, which is associated with falls and loss of functional independence. In a study of 1,200 older adults in Japan by Shimada et al. (2013), older adults with poorer performance in the Chair Stand Test were more likely to later be diagnosed with cognitive dysfunction or dementia than those with better performance. In a 6-year longitudinal study of 900 older adults, (Buchman et al. 2007) demonstrated that weaker lower limb muscle strength is associated with increased dementia incidence. Meanwhile, (Cui et al. 2021) indicate that grip strength is more strongly correlated with overall frailty and mortality risk than with cognitive dysfunction. The present findings therefore indicate that, although upper limb strength (as reflected by grip strength) may not differ markedly in early cognitive impairment, lower limb muscle strength appears to decline more sensitively during the early stages. Given its close relationship with gait stability, activities of daily living (ADL), and balance control, lower limb muscle strength may represent a particularly important functional marker in the early assessment of cognitive impairment. Notably, the observed difference demonstrated a large effect size, suggesting that lower limb functional performance may be particularly sensitive to early motor alterations associated with cognitive decline, despite the exploratory nature of the present study.

Third, in the balance analysis, no significant between-group differences were observed in ENV and RMS; however, total path length was significantly greater, whereas COP sway velocity was lower in the eMCI-W group. These findings suggest that, in early cognitive impairment, the overall spatial range of postural control may remain relatively preserved, while the characteristics of postural adjustments may differ. Specifically, although the total path length increased, the reduced sway velocity indicates slower but larger postural adjustments. COP sway velocity and total path length are considered indicators of sensorimotor integration efficiency and central nervous system control (Mancini et al., 2021; Ozga et al., 2023). The increased path length observed in the eMCI-W group may reflect greater postural excursions, while the reduced velocity may indicate a more cautious or constrained control strategy to maintain stability. By contrast, ENV and RMS are more sensitive to substantial impairments in balance capacity. In individuals with early-stage cognitive impairment, overall balance ability may still be sufficiently preserved to prevent detectable differences in these broader spatial indices. Taken together, these results suggest that early cognitive impairment may be associated not simply with increased instability, but with qualitative changes in postural control strategies. Therefore, when evaluating static balance, it is important to consider multiple COP-based variables rather than relying on a single indicator.

When the relationships between gait patterns and early cognitive impairment were analyzed, while basic gait parameters such as stride width showed no significant differences between groups, several spatiotemporal variables including stride length, gait cycle time, and double-limb support time were significantly altered in the eMCI-W group. These findings suggest that although overall gait performance appears relatively preserved, subtle alterations in gait timing and stability-related parameters may already be present in early cognitive impairment. This result is consistent with previous findings reported by (Ohsugi et al. 2019). Previous studies have suggested that gait speed tends to decline more prominently during later stages of dementia, whereas more subtle gait characteristics—such as variability, asymmetry, and temporal control—may be altered earlier in the disease process (Beauchet et al., 2019; Montero-Odasso et al., 2020; Del Din et al., 2021).

In particular, increased double-limb support time is commonly interpreted as a cautious gait strategy adopted to enhance stability during walking, especially in older adults with impaired balance control or increased fear of falling. Similarly, prolonged gait cycle time may reflect subtle alterations in gait timing and possible adaptive changes in motor-control behavior during walking. These interpretations are consistent with recent findings suggesting that subtle gait-control alterations and increased gait variability may be associated with cognitive impairment and fall-related risk in neurological populations (Dubbioso et al., 2023). From a clinical perspective, these alterations may reflect subtle changes in dynamic stability regulation during gait and postural regulation despite relatively preserved basic locomotor ability.

Previous studies have demonstrated that subtle gait instability and altered postural control associated with cognitive impairment may contribute to increased fall risk, mobility limitation, and functional decline in older adults with cognitive impairment and other neurodegenerative conditions (Amboni et al., 2013; Morris et al., 2016; Dubbioso et al., 2023).

Although, the present findings should be interpreted cautiously because of the exploratory pilot design, these results suggest that subtle gait and postural-control alterations may provide preliminary insight into mobility-related alterations in older adults with cognitive impairment.

In the analysis of angular velocity, most angular velocity measurements from the ankle and knee joints did not differ significantly between groups. However, the right hip showed significantly decreased angular velocity in the frontal plane (*Y*-axis) in the eMCI-W group (*p* < 0.001). Lateral angular velocity of the hip joint is known to be a key factor in trunk stability and fall prevention during gait (Mirelman et al., 2012). The present findings suggest that lateral stabilization strategies may already be compromised in the early stages of cognitive impairment. Similar to the ROM findings, there were no significant differences in the left hip or in other planes, further supporting the possibility that joint dysfunction in MCI presents in a selectively asymmetric manner. Taken together, these findings indicate that individuals in the eMCI-W group exhibit a considerable decline in lateral stabilization and control of the hip joint. Importantly, this variable also demonstrated a relatively large effect size, indicating that frontal-plane hip control may warrant further investigation in future longitudinal studies.

In summary, older women with eMCI-W showed relatively preserved body composition and basic gait ability. However, subtle alterations were observed in lower limb strength, fine control of static balance, and selective lower limb kinematic parameters. These findings suggest that, in early cognitive impairment, qualitative aspects of motor control such as balance regulation and joint stabilization may provide additional insight into subtle motor-control changes beyond global metrics such as gait speed. Thus, beyond the question of “How fast can you walk?,” clinical assessment approaches may also benefit from addressing “How well can you maintain balance and control your joints?” These qualitative motor-control characteristics of these variables may help characterize subtle motor-control features associated with early cognitive impairment (Montero-Odasso et al., 2020; Del Din et al., 2021; Mirelman et al., 2022).

It is important to note that this study was conducted as an exploratory pilot investigation with a relatively small sample size. Therefore, the possibility of both Type I and Type II errors cannot be excluded, particularly given the simultaneous analysis of multiple variables without formal correction for multiple comparisons. Accordingly, the findings should be interpreted as preliminary and hypothesis-generating rather than confirmatory evidence.

In addition, cognitive classification was based on MoCA screening rather than comprehensive clinical neuropsychological diagnosis, and the possibility of misclassification cannot be excluded. Furthermore, potential confounding variables—including physical activity level, medication use, depressive symptoms, comorbidities, and lifestyle factors—were not systematically controlled and may have influenced gait and balance outcomes.

Finally, because of the cross-sectional design, causal relationships between cognitive impairment and motor-control alterations cannot be established. Future studies should employ larger longitudinal designs incorporating formal clinical diagnosis, dual-task gait assessment, gait variability metrics, and fall-related outcomes to clarify the clinical significance of these preliminary findings. Therefore, the present findings should be interpreted as exploratory observations rather than definitive evidence of early motor biomarkers associated with cognitive impairment.

## 5 Conclusion

This study conducted an exploratory comparative analysis of differences in body composition, functional fitness, static balance, gait patterns, and lower limb ROM and angular velocity in older women with early cognitive impairment (eMCI-W) and healthy controls (HC-W).

- Older women with eMCI-W showed no significant differences in body composition compared to healthy controls.
- Lower limb muscle strength, as measured by the 30-second Chair Stand Test, was significantly reduced in the eMCI-W group.
- Static balance analysis revealed increased total COP path length and decreased sway velocity, indicating larger but slower postural adjustments.
- Several spatiotemporal gait parameters (stride length, gait cycle time, and double-limb support time) were significantly greater in the eMCI-W group.
- Selective reductions in knee joint ROM and hip joint angular velocity suggest subtle alterations in joint-level motor control.

Several limitations should be acknowledged. First, the small sample size limits generalizability. Second, potential confounding variables were not controlled. Third, the cross-sectional design limits causal inference. Future studies should employ longitudinal designs and incorporate advanced gait analyses to better understand motor–cognitive interactions.

## Data Availability

The datasets presented in this study can be found in online repositories. The names of the repository/repositories and accession number(s) can be found in the article/supplementary material.
